# *Pseudoclavibacter*-like subcutaneous infection: a case report

**DOI:** 10.1186/1752-1947-5-468

**Published:** 2011-09-20

**Authors:** François Lemaitre, Andreas Stein, Didier Raoult, Michel Drancourt

**Affiliations:** 1 Pôle des Maladies Infectieuses, Fédération de Microbiologie Clinique, Hôpital de la Timone, rue Saint-Pierre, 13005 Marseille, France; 2 Pôle des Maladies Infectieuses, Service de Maladies Infectieuses et Tropicales, Hôpital de la Conception, boulevard Baille, 13005 Marseille, France; 3 Unité de Recherche sur les Maladies Infectieuses et Tropicales Emergentes, CNRS-IRD UMR 6236, Faculté de Médecine, IFR 48, Université de la Méditerranée, 27 Boulevard Jean Moulin, F-13385 Marseille cedex 5, France

**Keywords:** *Pseudoclavibacter*, 16S rRNA gene, MALDI-TOF, identification, skin infection

## Abstract

**Background:**

*Arthrobacter*-like organisms, including *Pseudoclavibacter *organisms, have rarely been documented as being responsible for infection in humans.

**Case presentation:**

An 81-year-old French man developed a subcutaneous infection despite antibiotic treatment combining clindamycin and metronidazole for chronic wound infection. A skin biopsy showed numerous polymorphonuclear cells and no bacteria, but a subcutaneous swab yielded numerous polymorphonuclear cells, a few Gram-positive cocci, Gram-negative cocci, and Gram-positive rods. The Gram-positive rod sequence exhibited 99% sequence similarity with uncultured *Pseudoclavibacter *sp. [GenBank:EF419350] and 99% sequence similarity with uncultured *Pseudoclavibacter *sp. [GenBank:EF419347]. The genetic data and unique peptide profile of this *Pseudoclavibacter*-like isolate, determined by matrix-assisted laser desorption ionization-time of flight mass spectrometry, underscored its uniqueness.

**Conclusions:**

*Pseudoclavibacter*-like organisms are identifiable in cutaneous and subcutaneous infections in humans.

## Background

*Pseudoclavibacter *is an emerging bacterial genus created a few years ago to accommodate environmental *Brevibacterium *organisms [1]. Indeed, *Arthrobacter*-like bacteria have rarely been isolated in patients, and a *Pseudoclavibacter *organism has been reported to be isolated only once, from an aortic valve of a 74-year-old man [2].

## Case presentation

**Figure 1:**
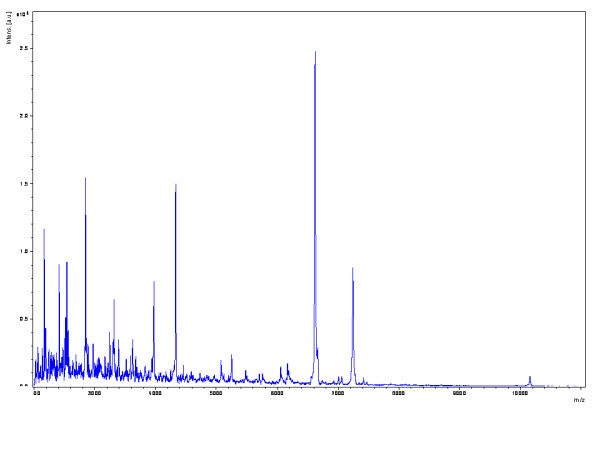
**Matrix-assisted laser desorption ionization-time of flight (MALDI-TOF) mass-spectrometry peptide profile of a *Pseudoclavibacter*-like organism**. This profile could be used as a reference for rapid identification of this bacterial species.

An 81-year-old French man was admitted to our hospital for erysipelas of the right leg. The patient had suddenly developed this infection despite antibiotic treatment combining clindamycin and metronidazole for a chronic wound infection of the same leg with previous documentation of clindamycin-susceptible *Staphylococcus aureus*, *Klebsiella oxytoca*, *Serratia marcescens*, and *Corynebacterium *spp., but no anaerobe. His leukocyte count was 10.32 g/L with 72% polymorphonuclear cells, 18% lymphocytes, and 8% monocytes. Inflammatory syndrome was apparent with a C-reactive protein level of 119 mmol/L and a fibrinogen level of 8.6 g/L. Antibodies against streptolysin O and streptococcal DNase were not detectable in the patient's serum. Direct microscopic examination of a skin biopsy showed numerous polymorphonuclear cells and no bacteria. Culture remained sterile after a five-day inoculation on Columbia agar with 5% sheep blood (bioMérieux, Marcy-l'Etoile, France), Chocolate agar with PolyViteX agar (bioMérieux) and MacConkey agar (bioMérieux) at 37°C in 5% CO_2_. Three days later a subcutaneous swab yielded numerous polymorphonuclear cells, and semi-quantitative direct examination indicated an average of 15 to 30 organisms per microscopic field composed of an equal proportion of Gram-positive cocci, Gram-negative cocci, and Gram-positive rods. Culture under the same conditions described above yielded small, gray colonies after 48-hour inoculation on Columbia agar with 5% sheep blood. The Gram-positive rod was oxidase-negative and catalase-positive. Inoculation of an API Coryne system identification strip (bioMérieux), performed twice, yielded no reaction and thus no identifying profile. No other organism was isolated from this specimen. Antibiotic susceptibility assessed on Mueller-Hinton agar (bioMérieux) using the disc method (Mast Diagnostics, Amiens, France) and break points as previously reported [3] yielded susceptibility to amoxicillin (minimum inhibitory concentration (MIC) ≤ 2 mg/L), rifampin (MIC ≤ 4 mg/L), doxycycline (MIC ≤ 4 mg/L), and vancomycin (MIC ≤ 4 mg/L) and resistance to co-trimoxazole, clindamycin, and metronidazole. The latter three antibiotics yielded no growth inhibition zone. To identify the isolate, we amplified (by polymerase chain reaction) and sequenced 1431 bases of the 16S rRNA gene [GenBank:FJ375951] [4]. This sequence exhibited 99% sequence similarity with uncultured *Pseudoclavibacter *sp. [GenBank:EF419350] and 99% sequence similarity with uncultured *Pseudoclavibacter *sp. [GenBank:EF419347]. The third hit exhibited only 97% sequence similarity with *Zimmermannella bifida *[GenBank:AB012589]. The peptide profile of the isolate was determined by matrix-assisted laser desorption ionization-time of flight (MALDI-TOF) mass spectrometry as previously described [5] (Figure 1). MALDI-TOF-based identification was achieved by comparing the isolate profile with the 3438 bacterial profiles deposited in the MALDI BioTyper database (Bruker Corp. Bremen, Germany), which includes 56 *Arthrobacter*, 17 *Brevibacterium*, three *Pseudoclavibacter*, and no *Zimmermannella *organism profiles (as of June 2010). The isolate was not identified with any of the species in the database, with the best identification score being 1.326 with *Corynebacterium afermentans*. The isolate has been deposited in the Collection de Souches de l'Unité des Rickettsies, Marseilles, France (CSUR P29). The clinical evolution was favorable under antibiotic treatment combining intravenous imipenem and vancomycin. Oral treatment with amoxicillin/clavulanate replaced intravenous antibiotics on day six.

Identification of the isolate was made possible only after 16S rRNA gene sequence analysis, as the isolate was not reactive on identification strips and did not exhibit any identifying phenotype. The 16S rRNA gene sequence comparison indicated that this isolate is representative of a previously uncultured organism. This case illustrates a new paradigm in using 16S rRNA gene sequence-based identification of organisms in clinical microbiology laboratories. Indeed, most of the emerging bacteria have been described previously on the basis of an original bacterial isolate exhibiting a 16S rRNA gene sequence with < 98.7% sequence similarity with any other sequence [4,6,7]. In our case report, however, the 16S rRNA gene sequence was already available in GenBank before we recovered the isolate. Indeed, extensive genomic and metagenomic explorations of complex environmental and mucosa-associated flora yielded a tremendous amount of the original 16S rRNA gene sequence from as-yet-uncultured organisms [8,9]. In our case report, isolation of an organism exhibiting a 16S rRNA gene sequence identical to that of a previously uncultured organism underscores the uniqueness of this isolate.

## Conclusions

In the case of our patient, the *Pseudoclavibacter*-like organism was most probably involved in his clinical infection, since this Gram-positive rod was observed in the presence of pus during the direct examination of a subcutaneous specimen. It grew in pure culture from a patient who was taking two antibiotics to which the *Pseudoclavibacter*-like organism was found to be resistant, thus supporting the hypothesis that growth of the *Pseudoclavibacter*-like organism was indeed selected by the antibiotic treatment. Also, *Pseudoclavibacter *sp. and other *Arthrobacter*-like organisms have never been reported as potential contaminants of culture, and *Pseudoclavibacter *spp. have not been isolated in our laboratory, with the exception of this patient. The 16S rRNA gene sequence of identical *Pseudoclavibacter*-like organisms was found in the diseased skin of patients with psoriasis before we obtained the first isolate [10]. This fact and the data presented in this case report suggest that *Pseudoclavibacter*-like organisms are organisms involved in skin diseases. *Pseudoclavibacter*-like organisms are bacterial organisms identifiable in cutaneous and subcutaneous infections in humans on the basis of a unique peptide profile obtained by MALDI-TOF analysis and unique 16S rRNA gene sequencing.

## Consent

Written informed consent was obtained from the patient for publication of this case report and any accompanying images. A copy of the written consent is available for review by the Editor-in-Chief of this journal.

## Competing interests

The authors declare that they have no competing interests.

## Authors' contributions

FL reviewed the medical and laboratory charts and was involved in drafting the manuscript. AS took care of the patient. DR and MD drafted the manuscript. All authors read and approved the final manuscript.
